# Bonnie Berger named ISCB 2019 ISCB Accomplishments by a Senior Scientist Award recipient

**DOI:** 10.1093/bioinformatics/btz389

**Published:** 2019-06-10

**Authors:** Christiana N Fogg, Ron Shamir, Diane E Kovats

**Affiliations:** Kensington, MD, USA; Computational Genomics Group, Blavatnik School of Computer Science, Tel Aviv University, Tel Aviv, Israel; International Society for Computational Biology, Leesburg, VA, USA

ISCB honors a leader in the fields of computational biology and bioinformatics each year with the Accomplishments by a Senior Scientist Award. This award is the highest honor conferred by ISCB to a scientist who is recognized for significant research, education and service contributions. Bonnie Berger, Simons Professor of Mathematics and Professor of Electrical Engineering and Computer Science at the Massachusetts Institute of Technology (MIT), is the 2019 recipient of the Accomplishments by a Senior Scientist Award. She is receiving her award and presenting a keynote address at the 2019 Joint International Conference on Intelligent Systems for Molecular Biology/European Conference on Computational Biology in Basel, Switzerland on July 21–25, 2019.

## Bonnie Berger: from math riddles to genomics

**Figure:**
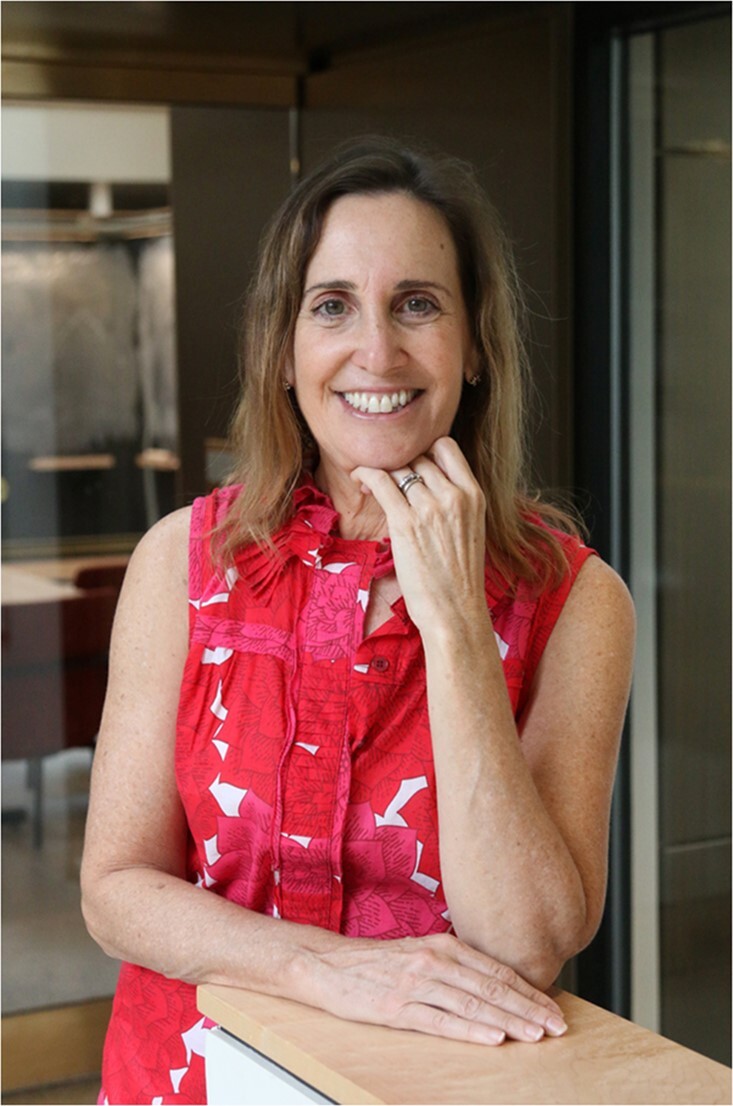


Bonnie Berger grew up in Miami, Florida with her parents and older brother and has early memories of being curious about mathematics. She recalled, ‘As a young child, I responded, ‘I want one too, please’, when my father slipped math problems under my brother’s door. My father would continue to challenge me with math riddles and chess puzzles. He would also engage me in science projects. Our relationship laid the foundation for my comfort with, and interest in, math and science, even though it was not so common for girls at the time’. Berger’s early interest in math and science led her to complete her AB in computer science at Brandeis University. In 1990, she completed her PhD in computer science at MIT under the mentorship of Silvio Micali. Berger’s dissertation research on randomized and parallel algorithms was recognized by the Machtey Award for a manuscript that she co-published with fellow graduate student John Rompel, as well as the George M. Sprowles Award.

After graduate school, Berger remained at MIT and stumbled upon computational biology quite unexpectedly. She recounted, ‘My postdoc supervisor Daniel J. Kleitman, who has Erdos #1 and solved dynamic programming for RNA base-pairings with Ruth Nussinov, had just come back from an NSF workshop whose goal was to get mathematicians and biologists together to solve challenges at the interface between the two fields. He was so taken with Michael Levitt’s talk that he said, ‘Proteins, that’s what you should do’. Well, fortunately, he didn’t say, ‘Plastics’, as in ‘The Graduate’, or I might have ended up a material scientist’. She appreciates the freedom she had as a postdoc and took to heart the advice Kleitman gave her when he told her, ‘We are applied mathematicians looking for interesting problems to investigate’. Following her postdoc, Berger became an Assistant Professor of Mathematics at MIT, and as a PI, she has pioneered the use of computer algorithms for analyzing, interpreting and sharing diverse types of biological data.

Among Berger’s scientific contributions, she developed the use of pairwise residue correlations to predict protein structure from sequence through her highly cited Paircoil/Multicoil programs. Her seminal work on the hardness of protein folding was recognized with the 2010 RECOMB Test of Time Award. Berger’s interest in genomics included development of the ARACHNE genome assembly tool, which was used by the Human Genome Consortium for whole genome assembly. She also initiated the area of comparative genomics with her cutting-edge work comparing human and mouse genomes. Berger launched the subfield of global network alignment with her Isorank/IsorankN programs and advanced protein structure alignment with her MATT program. More recently, Berger has founded the field of compressive genomics by designing algorithms that can be used for genomic analysis on compressed data in order to keep pace with data generation. She has also spearheaded efforts to improve biomedical data privacy, including the development of tools to securely crowdsource genomic and pharmacological data at scale.

Berger considers her theoretical computer science background to be critical to her success in identifying and studying computational biology problems. She said, ‘I have realized that with my algorithms background and flexibility, I can easily shift between areas as the research landscape changes. As I gain knowledge across diverse research areas, I can see connections between them and techniques that can be used to address them’. Berger has also come to appreciate the many mentors that helped her bridge the gap between computer science and biology, including Peter Shor, Peter S. Kim and Jonathan King. She recalled, ‘[They] taught me biology on a need-to-know basis. It took many rounds of back-and-forth by, would you believe, fax machine with Peter Kim for me to turn my early Paircoil writeup from definitions and theorems to one accessible to a biology audience’.

Berger has trained numerous graduate students and postdocs, using an approach she learned from her NSF Postdoc supervisor. She said, ‘[Kleitman] gave me a lot of freedom to pursue whatever interested me, and that’s how I mentor my students. I ask them what interests them and suggest several research problems, or I encourage them to bring entirely new research areas to us’. Many of her trainees have become leaders in the field of computational biology.

Berger is also fascinated by developing methods to improve data sharing and said, ‘I am interested in individuals, labs and companies owning rights to their own data but providing provably secure algorithms so that they can for the first time share their data at scaleto enable biomedical insights across different nations and diverse data. Recently, I am interested in developing sketching algorithms that take advantage of the geometry of single-cell RNA-seq data to better share, analyze and draw insights from the data’.

Berger has served the computational biology community in many capacities, including her roles as Vice President of ISCB and Head of the RECOMB Steering Committee; as well as her service on multiple editorial boards, and program and conference committees. Her scientific contributions have been recognized by numerous awards, including the NSF Career Award, Biophysical Society Dayhoff Award for Research, inaugural Technology Review Top 100 Innovators, ACM Fellow, ISCB Fellow, AMS Fellow, AIMBE Fellow, NIH Margaret Pittman Award for Outstanding Scientific Achievement & Lectureship, election to the American Academy of Arts & Sciences and an Honorary Doctorate from EPFL.

Berger is extremely grateful for this recognition by ISCB, especially considering her longtime involvement with the Society. She said, ‘It’s a tremendous honor to join such a distinguished and accomplished group of scientists’.

